# Dr. Daniel Neall

**Published:** 1894-02

**Authors:** 


### Obituary.

### DR. DANIEL NEALL.

Daniel Neall departed from this life January 6, 1894, aged seventy-seven years.

The gradual passing from the active scenes of earth of many of the earliest workers in dentistry should lead those who are left to entertain the serious reflection that the bonds which have for so long a period united this generation with the past are being rapidly severed.

Daniel Neall was contemporary with Maynard, Harris, Westcott, Arthur, Townsend, and the untiring host of original thinkers who made dentistry what it is to-day. He labored with all of these that it should be assured of a foundation upon which to erect a superstructure honorable to all who practised it. While he thus actively worked he shunned publicity, preferring modest retirement. Yet his influence was deeply felt in the professional circle in which he moved.

He came from sturdy, uncompromising Quaker stock. His father began the practice of dentistry when the subject of this sketch was quite a boy, and consequently it may be truly said that his entire business life was spent in dental work.

Daniel Neall, Sr., was among the earliest of the practitioners in Philadelphia, and while Plantou has the credit of introducing porcelain teeth in America in that city, Dr. Neall, the elder, greatly improved on the work. It was the writer's privilege to see a good deal of his block-work as early as 1837, and, while markedly inferior to that of Wildman of a later period, it effectively accomplished the purpose intended.

The son inherited a taste for the mechanical side of the profession, but devoted himself principally to the operative branch, in which he excelled. While his work was marked with skill, it is questionable whether he ever fully adopted modern methods. He was strictly an operator of the old school, but one of the very best in his class, and few could excel him at the present time.

He early became a member of the Pennsylvania Association of Dental Surgeons, and was an active participant in its proceedings. He was also an active member in the Odontological Society of Pennsylvania.

For some years he was a member of the Board of Trustees of the Pennsylvania College of Dental Surgery. While, theoretically, an earnest advocate for advanced educational methods, with this exception he abstained from active participation in the work. His view, if the writer understood him correctly, was, that a man in active practice could not teach and do justice to his patients. He was conservative in many directions and broad in others,—a very common condition with men of positive temperament.

Very few persons possessed greater conversational powers. He was the life of the social circle, and his membership with the Philadelphia Dental Club, a social organization, will long be remembered by those connected with it.

His health was much broken for several years, preventing him from practice, but when he returned to it he probably found but little change in the number of his clientele. The devotion shown to him is rare, but in this case it was founded on a true love for the man.

His funeral was largely attended, and, doubtless, as he wished, in the quiet, unostentatious manner peculiar to the religious society in which he had been educated.

Thus passed from active life one of the best examples of the early members of the dental profession, and we who knew his many sterling qualities, his unswerving honesty, and the constant exhibition of the highest manhood, must ever mourn his loss.

For several years his health has been such as to force him to abandon all practice, but the true exponent of a higher professional life and spirit can never be forgotten, and such a one was Daniel Neall.

